# Nuclear and cytoplasmic expression of survivin in 67 surgically resected pancreatic cancer patients

**DOI:** 10.1038/sj.bjc.6602632

**Published:** 2005-05-31

**Authors:** G Tonini, B Vincenzi, D Santini, S Scarpa, T Vasaturo, C Malacrino, R Coppola, P Magistrelli, D Borzomati, A Baldi, A Antinori, M Caricato, G Nuzzo, A Picciocchi

**Affiliations:** 1 Medical Oncology, University Campus Bio-Medico, Rome, Via Emilio Longoni 83, 00155 Rome, Italy; 2 Department of Experimental Medicine and Pathology, University ‘La Sapienza’, Rome, Italy; 3 Department of Surgery, University Campus Bio-Medico, Rome, Italy; 4 Department of Surgery, Catholic University School of Medicine, Rome, Italy; 5 Department of Biochemistry and Biophysics, ‘F Cedrangolo’, Section of Anatomic Pathology, Second University of Naples, Naples, Italy

**Keywords:** pancreatic carcinoma, surviving, Cox-2, prognosis, apoptosis

## Abstract

Pancreatic cancer is one of the most aggressive gastrointestinal cancer with less than 10% long-term survivors. The apoptotic pathway deregulation is a postulated mechanism of carcinogenesis of this tumour. The present study investigated the prognostic role of apoptosis and apoptosis-involved proteins in a series of surgically resected pancreatic cancer patients. All patients affected by pancreatic adenocarcinoma and treated with surgical resection from 1988 to 2003 were considered for the study. Patients' clinical data and pathological tumour features were recorded. Survivin and Cox-2 expression were evaluated by immunohistochemical staining. Apoptotic cells were identified using the TUNEL method. Tumour specimen of 67 resected patients was included in the study. By univariate analysis, survival was influenced by Survivin overexpression. The nuclear Survivin overexpression was associated with better prognosis (*P*=0.0009), while its cytoplasmic overexpression resulted a negative prognostic factor (*P*=0.0127). Also, the apoptotic index was a statistically significant prognostic factor in a univariate model (*P*=0.0142). By a multivariate Cox regression analysis, both the nuclear (*P*=0.002) and cytoplasmic (*P*=0.040) Survivin overexpression maintained the prognostic statistical value. This is the first study reporting a statistical significant prognostic relevance of nuclear and cytoplasmic Survivin overexpression in pancreatic cancer. In particular, patients with high nuclear Survivin staining showed a longer survival, whereas patients with high cytoplasmic Survivin staining had a shorter overall survival.

Pancreatic duct cell carcinoma (PDC) is one of the most malignant gastrointestinal tumours. Once PDC is clinically evident, it progresses rapidly to develop metastatic lesions and this event often occurs by the time of diagnosis. Furthermore, this tumour is usually resistant to conventional chemotherapy and radiation therapy. The pathogenic mechanisms that regulate this aggressive growth behavior of PDC still is to be clarified (Satoh *et al*, 2001).

Apoptosis or programmed cell death plays a critical role in normal morphogenesis and homeostatic mechanisms in both normal and neoplastic cells. The suppression of apoptosis, by aberrantly prolonging cell viability, is considered to contribute to carcinogenesis and carcinoma progression by facilitating gene mutations and promoting resistance to immune-based cytotoxicity (Thompson, 1995).

Apoptosis is implemented by a family of cysteine proteases known as caspases. These are produced inside the cell as inactive zymogens and generally must undergo proteolytic processing to become active proteases (Jaattela, 1999; Nicholson, 1999).

The inhibitors of apoptosis protein (IAPs) are the only known endogenous caspase inhibitors (Deveraux *et al*, 1997). They contain *Baculovirus* IAP repeat domains, and some of them bind and potently inhibit activated caspases, including in mammals the effector caspases-3 and -7 and the initiator caspase-9 (Deveraux and Reed, 1999).

In addition to *Baculovirus* IAP repeat domains, several IAPs also contain a RING domain, which binds ubiquitin-conjugating enzymes that promote degradation of IAP caspase complexes (Yang *et al*, 2000).

Survivin is a member of IAPs family and contains a single baculovirus IAP repeat (Ambrosini *et al*, 1997; LaCasse *et al*, 1998). It is expressed during human foetal development, but not detectable in normal adult tissues, except in thymus and placenta. Its expression has been showed in several neoplasms, including pancreatic, gastric, colonic, lung, breast, prostatic and bladder cancers, neuroblastomas, and lymphomas (Adida *et al*, 1998a; Ambrosini *et al*, 1998; Kawasaki *et al*, 1998; Lu *et al*, 1998; Swana *et al*, 1999; Kobayashi *et al*, 1999).

For these malignancies, previous studies established that Survivin is an important mediator in carcinogenesis; it acts as a resistance factor by inhibiting caspase activity in cells exposed to a variety of genotoxic stresses such as anticancer drugs and Fas-ligand (Li *et al*, 1998, 1999; Tamm *et al*, 1998).

The prognostic role of apoptosis, pro- and antiapoptotic molecules in human cancer is still a debating matter: previous studies suggested that the Survivin expression in cancer is associated with a more aggressive disease and with shorter patients survival (Li, 2003). However, it must be pointed out that besides the increase of published papers about Survivin in the last 3 years, inconsistent observations have been reported on this item (Li, 2003).

Other molecules supposed to be involved in the modulation process of apoptosis in human solid cancer have been investigated and some of them are tightly related with Survivin. Recently, Krysan *et al* have demonstrated that the overexpression of Cox-2 significantly increases the survival of NSCLC cells exposed to apoptotic stimuli and that the expression of antiapoptotic protein Survivin has correlated positively correlates with the Cox-2 expression. As a consequence, the authors suggested that in the Cox-2 overexpressing cells, Survivin is stabilised due to the lowered ubiquitination levels, which may account for the elevated apoptosis resistance of these cells (Krysan *et al*, 2004).

The aim of the present study was to investigate the prognostic role of Survivin and Cox-2 expression and the modulation of their apoptotic pathway in a series of 67 pancreatic cancer patients treated with radical surgical resection. The impact of the Survivin cellular distribution (nuclear *vs* cytoplasmic) on prognosis in this cohort of patients was also evaluated.

## PATIENTS AND METHODS

### Clinical data and tumour specimen acquisition

In order to obtain the most possible consistent and homogeneous group of patients, only patients with no macroscopic residual tumour were considered, were treated at the Catholic University School of Medicine of Rome and at the University Campus Bio-Medico of Rome from January 1986 through April 2003. Patients were staged before surgery by CT-Scan of the thorax, abdomen and pelvis. If necessary, intraoperative ultrasound of the liver was performed. Intraoperative staging always confirmed the absence of distant metastases and of infiltration of mesenteric vessels and/or portal vein. Preoperative staging showed a tumour of the pancreatic head in 54 cases (84.7%), of the body and/or of the tail in six cases (8.9%) and a diffuse neoplasm in seven (10.4%). All patients underwent surgical resection with standard lymphadenectomy. All patients affected by diffuse tumour underwent total pancreatectomy. In the presence of a body and/or tail tumour, distal pancreatectomy was always performed. Finally, in case of a cephalic tumour, pancreatoduodenectomy was carried out (Whipple: 14 cases, pylorus-preserving: 40 cases). Exclusion criteria for our analyses were perioperative mortality and the presence of macroscopic residual disease after resection.

Data on clinical parameters, including sex, age, preoperative assessment of disease state and type of operative procedure, were gathered retrospectively from patient records. Pathologic findings (tumour size, tumour location, involvement of surrounding structures and lymph node status) were obtained from the pathologists' original reports. In addition to the original pathology reports, microscopic findings (tumour type, degree of differentiation and TNM classification) were reassessed.

Tumours were categorised as International Union Against Cancer (Sobin and Wittekind, 2002).

Survival was determined from the date of initial surgery. Follow-up was available for all patients. Subjects who died because for causes other than pancreatic cancer during the follow-up period were considered for survival analysis.

### Histology

The formalin-fixed, paraffin-embedded samples were sectioned at 5 *μ*m and stained with haematoxylin and eosin. The histological diagnosis was re-examined. In addition, the most representative blocks were selected to be cut into 5 new *μ*m-thick sections for immunohistochemical studies.

### Immunohistochemistry and quantification of the immunoreactivity

Immunohistochemical studies were performed on 5-mm sections by a streptavidin-biotin-peroxidase system using a commercial kit (UCS Diagnostic, West Logan, UT, USA), according to the manufacturer's instructions. In brief, sections were de-paraffinised and antigen retrieval was achieved by steaming slides for 35 min in citrate buffer pH 6. Endogenous peroxidase was blocked using 3% hydrogen peroxide solution in PBS for 5 min. The following primary antibodies were used: rabbit polyclonal anti-Survivin (ABcam, Cambridge, UK), goat polyclonal anti-Cox-2 (Santa Cruz Biotechnologies, Santa Cruz, CA, USA). Sections were counterstained with haematoxylin and mounted. The primary antibody was replaced with rabbit or goat preimmune serum as a negative control for nonspecific staining. The stained sections were observed using a light microscope, and positivity was determined by cell staining.

Antigens were quantified according to the following two parameters: (1) the number of positively stained cells; (2) the intensity of the staining, ranging from pale pink to dark orange. Therefore, Cox-2 and Survivin positivity was graded on the basis of the intensity and the number of positive cells: 0: negative; +1: weak to moderate positive affecting less than 50% of the tumour area; +2: weak to moderate positive on the majority of the tumour or strong positive in the minority of the tumour; and +3: strong positive in the majority of the tumour area. The specimens with a grade amounting to more than +1 were regarded as positive, and 0 grade as negative. These scores were performed in a blinded fashion.

### Detection of apoptosis

Apoptotic cells were identified by the terminal deoxynucleotidyl transferase (TdT)-mediated deoxyuridine triphosphate biotin nick-end labelling (TUNEL) method. Dewaxed and rehydrated specimens were incubated in proteinase K 40 *μ*g ml^−1^ for 1 h at 37°C and were treated with 3% H_2_O_2_ in methanol for 30 min at room temperature. After adding equilibration buffer for 5 min at room temperature, TdT enzyme was pipetted onto the sections and incubated at 37°C for 2 h. The reaction was stopped by incubating the sections in stop buffer for 30 min at 37°C. Antidigoxigenin peroxidase was added to the slides, followed by incubation for 30 min at 37°C. Slides were stained with diaminobenzine for 10 min and counterstained with haematoxylin. A total of 500 cells were counted in each specimen. The apoptotic index was defined as follows: apoptotic index (%)=100 × apoptotic cells/total cells. We stratified tumour specimens according to TUNEL staining in <10% or >10% stained cells.

### Statistical analysis

The Spearman correlation test was used to assess the relationship between original ordinal data before binary re-categorisations (correlation matrix between immunostaining parameters). A univariate survival analysis for each prognostic variable on overall survival was estimated according to the Kaplan–Meier method (Kaplan and Meier, 1958). The terminal event was death attributable to cancer or noncancer causes. The statistical significance of the differences in survival distribution among the prognostic groups was evaluated by the log-rank test (Peto *et al*, 1977). The Cox proportional hazards model was applied to the multivariate survival analysis (Cox, 1972). The prognostic variables on overall survival included age, gender, T factor, N factor, adjuvant therapy, TUNEL staining, cytoplamatic and nuclear Survivin expression and Cox-2 staining. *P*-values <0.05 was regarded as statistical significant in two-tailed tests. SPSS software (version 10.00, SPSS, Chicago) was used for statistical analysis.

## RESULTS

### Patients' characteristics

The cohort (Table 1) consisted of 67 patients with the diagnosis of PDC (45 men and 22 women). All patients underwent surgical resection of the tumour. The median age at diagnosis was 63 years (range 45–83). Histopathological tumours features are summarised in Table 1.

Median follow-up after surgery was 22 months (range: 3–100 months). The minimum follow-up for patients without tumour recurrence was 9 months. In total, 14 patients were still alive at census-taking (July 2004).

Of the 67 patients, 42 (93.3%) died of pancreatic cancer and three (6.7%) of other causes. No patient was lost during the follow-up.

The overall median survival time was 18.5 months (range: 3–92 months). The overall 1-year disease-specific survival rate was 76.2%, with a 5-year survival rate of 22.8%.

Adjuvant therapy has not been routinely offered in the hospitals involved in the study. We identified 28 (41.8%) of 67 patients who received any form of adjuvant chemotherapy within 3 months of their operation and 19 patients (28.4%) who received adjuvant radiotherapy. Chemoradiation was administered in 11 patients (17.5%). No patients were treated with preoperative concomitant chemoradiation.

Protocols for chemotherapy were not standardised, but chemotherapy was 5-fluorouracil or gemcitabine-based.

### Cox-2, Survivin and TUNEL staining

Table 2 presents summary results from immunohistochemical analysis of the 67 patients included in the study. The expression analysis of Survivin revealed that in 42 out of 67 (62.7%) specimens, no nuclear expression was recorded, while in 37 (55.2%) specimens the cytoplasmic staining for Survivin was negative.

**Figure 1:**
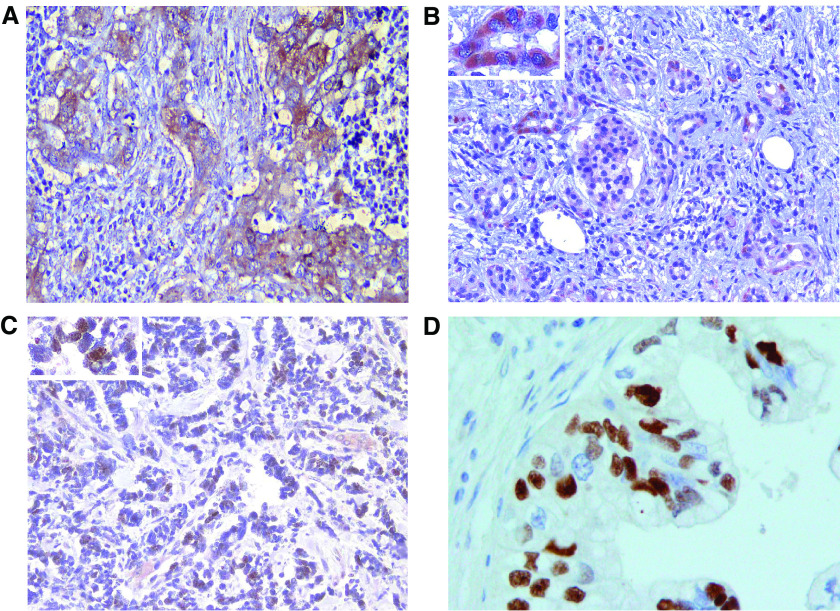
(**A**) Cytoplasmic positivity of immunohistochemical staining of Cox-2 (× 40); (**B**) cytoplasmic positivity of immunohistochemical staining of Survivin (× 20), with an enlarged particular showing the peculiar cytoplasmic staining; (**C**) nuclear positivity of immunohistochemical staining of Survivin (× 20) with an enlarged particular showing the peculiar nuclear staining; (**D**) TUNEL nuclear positive staining (× 400).

Moreover, 32 (47.8%) cases were considered as positive for Cox-2 staining. Cox-2 expression was always cytoplasmic. In 28 (41.8%) pancreatic cancer specimens, TUNEL staining was present in more than 10% of the observed cancer cells. Figure 1 shows the pictures of immunohistochemical staining for the molecular markers investigated in the present paper.

### Immunohistochemical and clinico-pathological parameters and patient survival

According to our analysis, univariate analysis showed that overall survival is influenced by the Survivin expression and cellular distribution. In particular, those patients with positive staining for nuclear Survivin showed longer overall survival than those with negative nuclear Survivin expression (10.00 *vs* 27.00; *P*=0.0009).

On the contrary, patients with cytoplasmic Survivin expression revealed a statistically significant shorter overall survival time when compared with those with negative staining (10.00 *vs* 25.00; *P*=0.0127). The median survival time in patients with low apoptotic index, evaluated by the TUNEL method, was 20.00 *vs* 8.00 months in those with high index (*P*=0.0142), while Cox-2 staining did not influence the overall survival time (Table 3).

**Figure 2:**
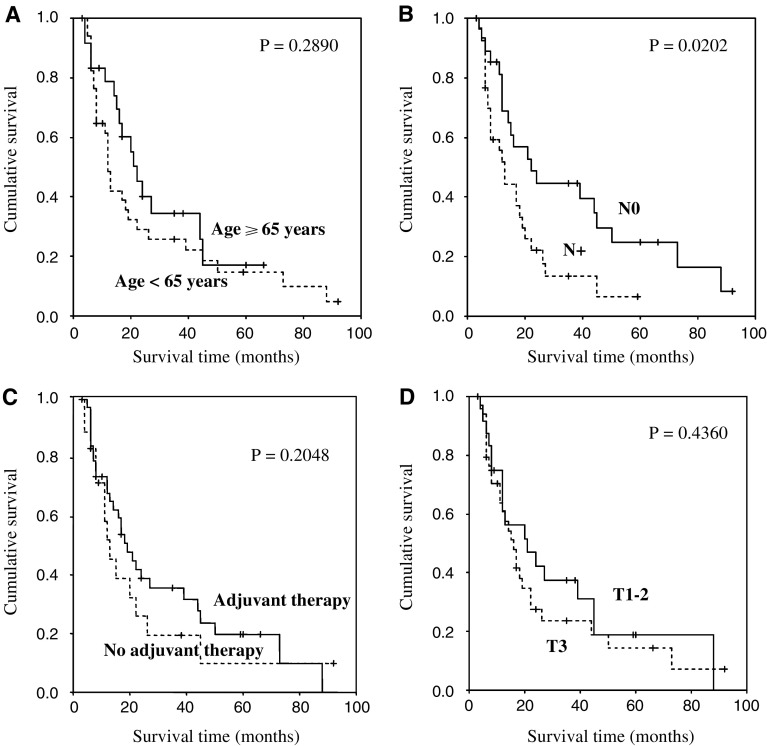
Kaplan–Meier survival curves for radically resected pancreatic cancer patients: (**A**) age (<65 years *vs* > 65 years); (**B**) N stage (nodal involvement *vs* no nodal involvement); (**C**) adjuvant therapy (any adjuvant therapy *vs* no adjuvant therapy); (**D**) T stage (T1–2 *vs* T3).

Figure 2 includes Kaplan–Meier survival plots in relation to clinico-pathologic patients' features. The only parameter that significantly correlated with overall survival time was the presence of metastatic lymph nodes (*P*=0.0202). Adjuvant therapy did not show any influence on overall survival (any adjuvant therapy: *P*=0.2048; postoperative chemotherapy: *P*=0.4790; radiotherapy: *P*=0.1102). However, those patients who received chemoradiation as adjuvant therapy after resection showed a longer median overall survival (19.00 months) than those patients who did not (13.00 months), even if this difference does not reach a statistical significance (*P*=0.0960). Probably, this non statistically significant difference is due to the small number of patients who received chemoradiation.

**Figure 3:**
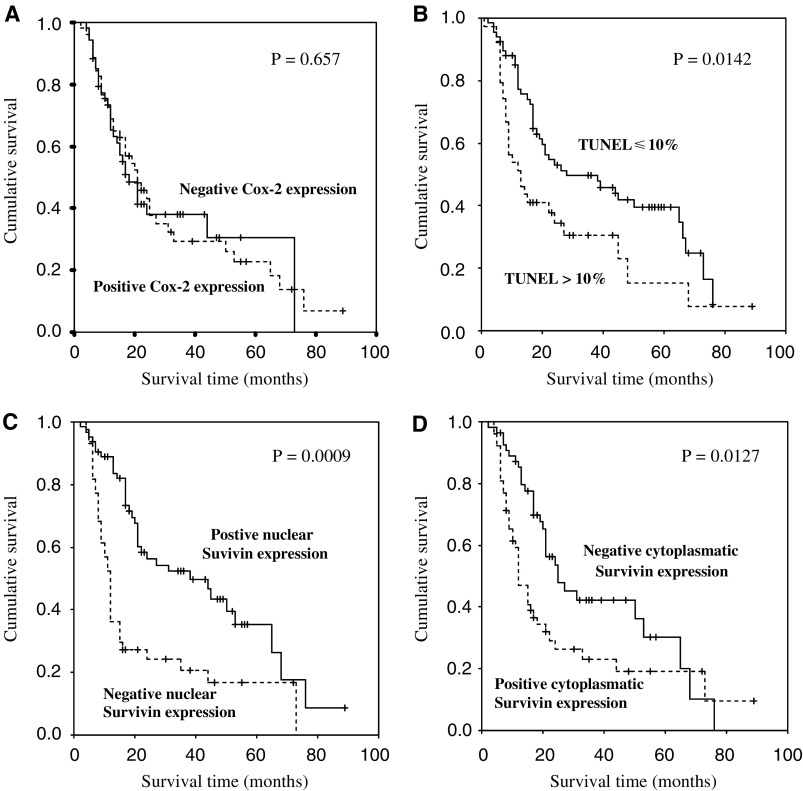
Kaplan–Meier survival curves for radically resected pancreatic cancer patients: (**A**) Cox-2 expression (positive Cox-2 expression *vs* negative Cox-2 expression); (**B**) TUNEL staining (>10 *vs* <10%); (**C**) nuclear Survivin expression (positive nuclear Survivin expression *vs* negative nuclear Survivin expression); (**D**) cytoplasmatic Survivin expression (positive cytoplasmatic Survivin expression *vs* negative cytoplasmatic Survivin expression).

Figure 3 depicts Kaplan–Meier survival plots for all patients showing the relation between either Cox-2 staining (A), the apoptotic index (B), nuclear (C) and cytoplasmic (D) Survivin expression and clinical outcome.

By a multivariate Cox regression analysis, the only immunohistochemical parameter that significantly influenced overall survival was the Survivin expression by cells. Both nuclear and cytoplasmic expression of Survivin resulted statistically significant prognostic factors at the multivariate analysis. The calculated relative risk in patients with positive nuclear staining was lower than in patients with negative staining (0.430; *P*=0.002). However, the positive cytoplasmic staining for Survivin in this cohort of patients was a statistically significant negative prognostic factor. In particular, the relative risk in patients with negative Survivin staining was 0.056 when compared with the risk of patients with positive cytoplasmic staining patients (*P*=0.040) (see Table 4).

## DISCUSSION

Pancreatic cancer is a very aggressive neoplasm with very poor prognosis. Surgical treatment is the only therapeutic option potentially able to cure this tumour. However, in more than 80% of cases showing pancreatic cancer, patients cannot undergo surgical treatment because of clinically advanced disease. Nonsurgical treatments offer little, if any, survival advantage. As a result, mortality almost parallels its incidence, with a 5-year survival rate of less than 10% (Kimura *et al*, 1998). There are only few substantial data reporting clinical significant prognostic markers for pancreatic cancer patients. Sohn *et al* (2000) showed by multivariate analysis that negative resection margins, tumour size and differentiation were important independent prognostic indicators. Similarly, Geer and Brennan (1993) demonstrated prognostic significance of tumour size, differentiation and lymph node involvement in both univariate and multivariate analyses. Nitecki *et al* (1995) from the Mayo Clinic showed that 5-year survival was significantly greater for node-negative *vs* node-positive patients (14 *vs* 1%), and for patients with smaller tumours *vs* patients with larger tumours (20 *vs* 1%). Moreover, in this paper, a combination of node-negativity and lack of perineural or duodenal invasion constituted a significant prognostic marker (Nitecki *et al*, 1995). Finally, Kawesha *et al* (2000) demonstrated that significant prognostic factors in pancreatic cancer patients were TNM stage of disease and lymph node involvement.

In addition, preoperative estimation of tumour size and lymph node involvement is difficult. As a consequence, patient selection for surgical resection based on preoperative estimation of these parameters often results inappropriate.

Much more interest is now focused on the role of molecular markers to select patients with better prognosis and those who could benefit of more aggressive treatments. Dysregulation of the normal cell-cycle regulatory machinery and of apoptosis mechanisms are integral to the neoplastic process, and there is now compelling evidence implicating loss of cell-cycle control in the development and progression of most human cancers (Sherr, 1996).

In the present paper we investigated the prognostic role of Survivin overexpression, Survivin cellular localisation and Cox-2 staining in a uniform cohort of patients affected by pancreatic cancer treated with radical surgery. These factors have been evaluated in conjunction with TUNEL staining for the detection of apoptotic cells.

In our analysis we identified for the first time that a different prognostic role is played by the nuclear and cytoplasmic Survivin expression. In particular, the nuclear expression of Survivin identified patients with a good prognosis, while the cytoplasmic overexpression of Survivin is a negative prognostic factor. These findings have been confirmed by univariate and multivariate analysis of survival.

There are some conflicting data published about the role of Survivin overexpression in cancer patients.

Cytoplasmic Survivin immunoreactivity has been observed in the vast majority of human tumours and it has been constantly associated with poor prognosis (Altieri *et al*, 1999).

Previous data suggested that Survivin is accumulated in advanced tumour stages, thus suggesting that its expression tends to increase with tumour progression. This evidence could justify the poor prognosis of human cancer with high cytoplasmic Survivin expression (Adida *et al*, 1998b; Swana *et al*, 1999; Ito *et al*, 2000).

Scarce data are available regarding Survivin nuclear localisation in human tumours. Interestingly, nuclear Survivin localisation in gastric (Okada *et al*, 2001) and transitional cell carcinoma (Lehner *et al*, 2002) is considered predictive of a favourable prognosis. Recently, Survivin has been detected in the nucleus of non-small-cell lung cancer cells from clinical samples, without any significant relation with clinical outcome (Falleni *et al*, 2003).

Finally, in two recent papers, nuclear Survivin overexpression has been associated with poor prognosis in mantle cell lymphoma (Martinez *et al*, 2004) and oesophageal squamous cell carcinoma (Grabowski *et al*, 2003). The results of these studies contributed to the confusion regarding the role of nuclear expression of Survivin. The reason for the different subcellular location of Survivin in different cancers is unclear. A recent report by Fortugno *et al* (2002) showed that Survivin exists in two distinct subcellular pools (cytoplasm and nucleus). The two Survivin pools are immunochemically distinct and independently modulated during cell cycle progression. The immunochemical differences between nuclear and cytosolic Survivin may explain, in part, the conflicting data about Survivin localisation reported in the literature and its prognostic role. In fact, two regions in Survivin that exhibit strikingly differential antibody reactivity were identified by Fortugno *et al* (2002): Cys^57^-Trp^67^, which is exposed in cytosolic and centrosome-associated survivin, but masked in nuclear and microtubulebound survivin, and Ala^3^-Ile^19^, which is accessible in kinetochore-associated Survivin, but not in the cytosolic form. A plausible interpretation of these data is that separate post-translational modifications may differentially affect epitope accessibility of nuclear *vs* cytosolic/microtubule-bound Survivin *in vivo*.

The mechanisms for the shift in the intracellular distribution of Survivin and its nuclear translocation in human cancer cells are still unclear. Suzuki *et al* found that the nuclear translocation in HepG2 cells is dependent both on Fas stimulation and cell proliferation. Survivin interacts with Cdk4 on translocation to nucleus, which releases p21 from the cdk4/p21 complex, forming a procaspase 3/p21 complex that resists Fas-mediated cell death (Suzuki *et al*, 2000). Rodriguez *et al* proposed that the subcellular distribution of survivin is regulated by an active import into the nucleus and a CRM1-mediated export to the cytoplasm, suggesting that survivin may be considered a nuclear shuttling protein. Predominantly cytosolic localisation in a high number of tumours may be the result of a high rate of nuclear export (Rodriguez *et al*, 2002).

Our study also explored the prognostic role of Cox-2 in our patients. Recently, Krysan *et al* (2004) have reported a frequent coexpression of Cox-2 and Survivin in non-small-cell lung cancer patients. As a consequence of these findings, we explored the prognostic impact of Cox-2 in pancreatic cancer patients in association with Survivin overexpression. Our findings do not suggest any role of Cox-2 in determining the clinical outcome in radically resected pancreatic cancer patients.

Finally, we evaluated the prognostic role of apoptotic index. Our investigation revealed that patients with higher apoptotic index benefit of a longer median survival time if compared with those with lower apoptotic index. However, when evaluated in multivariate analysis, the apoptotic index did not maintain a statistical significant value on prognosis. Only few data are available about the prognostic role of the apoptotic index in pancreatic cancer patients. Sarela *et al* (2002) failed to identify any prognostic relevance of apoptotic index in pancreatic adenocarcinoma patients (advanced or radically resected). Separately, Nio *et al* (2001) confirmed that apoptotic index has not a prognostic role in a cohort of 66 radically resected pancreatic cancer patients.

In conclusion, the present study is the first report that established the prognostic relevance of the Survivin expression in pancreatic cancer in relation with its cellular distribution. The knowledge of the factors that have an independent influence on prognosis is crucial for the development and interpretation of prospective randomised trials in which radically operated pancreatic cancer patients are stratified and treated with adjuvant therapies according to these prognostic determinants.

## Figures and Tables

**Table 1:** Patients' characteristics

Total number	67
Median age (range)	63 (45–83) years

*Gender*	
Male *vs* female	45 *vs* 22 (67.2 *vs* 32.8%)

*Pancreatic cancer site*	
Head	54 (80.6%)
Tail/body	6 (9.0%)
Diffuse	7 (10.4%)

*Factor*	
T1	8 (11.9%)
T2	17 (25.4%)
T3	40 (59.7%)

*N factor*	
Negative	33 (49.3%)
Positive	34 (50.7%)

*Grading*	
Well differentiated	14 (20.9%)
Moderate differentiated	28 (41.8%)
Poor differentiated	15 (22.3%)

*Postoperative radiotherapy*	
Yes	19 (28.4%)
No	48 (71.6%)

*Postoperative chemotherapy*	
Yes	28 (41.8%)
No	39 (58.2%)

*Postoperative chemoradiation*	
Yes	11 (16.4%)
No	56 (83.6%)
Median follow-up time (median; range)	12 (7–112) months
Median overall survival (median; range)	18.5 (3–98) months

**Table 2:** Immunohistochemical parameters pancreatic adenocarcinoma patients

	**Number**	**%**
Nuclear Survivin expression		
Survivin negative	42	62.7
Survivin positive	25	37.3

*Nuclear survivin expression*		
Survivin negative	37	55.2
Survivin positive	30	44.8

*Cox-2 expression*		
Cox-2 negative	35	52.2
Cox-2 positive	32	47.8

*TUNEL staining*		
TUNEL <10	39	58.2
TUNEL >10	28	41.8

**Table 3:** Univariate analysis of survival in radically operated pancreatic adenocarcinoma patients

	**Median survival (months)**	**95% CI**	***P*-value**
*Gender*			
Female	13.00	7.99–18.01	0.4020
Male	19.00	13.40–24.60	

*Age*			
<65 years	12.00	10.23–13.77	0.2890
>65 years	22.00	16.36–27.64	

*T factor*			
T1–2	21.00	4.71–37.29	0.4360
T3	16.00	11.67–20.33	

*N factor*			
N0	24.00	9.12–34.88	0.0202
N+	13.00	9.70–16.30	

*Adjuvant therapy*			
No adjuvant therapy	15.00	12.33–19.34	*P*=0.2048
Any adjuvant therapy	22.00	13.67–28.11	

*Nuclear Survivin expression*			
Survivin negative	10.00	10.34–13.66	0.0009
Survivin positive	27.00	20.03–31.97	

*Cytoplasmic Survivin expression*			
Survivin negative	25.00	8.87–29.66	0.0127
Survivin positive	10.00	7.34–17.65	

*Cox-2 expression*			
Cox-2 negative	18.00	8.07–27.93	0.657
Cox-2 positive	17.00	10.83–23.17	

*TUNEL staining*			
TUNEL <10	24.00	14.42–25.58	0.0142
TUNEL >10	11.00	2.55–13.45	

**Table 4:** Multivariate analysis of survival in radically operated pancreatic adenocarcinoma patients

	**Relative risk**	**95% CI**	***P***
*N factor*			
N+	1	—	0.145
N0	0.745	0.363–1.160	

*Nuclear Survivin expression*			
Survivin negative	1	0.211–0.897	0.002
Survivin positive	0.430		

*Cytoplasmic Survivin expression*			
Survivin positive	1	0.325–0.901	0.040
Survivin negative	0.556		

*TUNEL staining*			
TUNEL>10	1	0.451–1.430	0.105
TUNEL <10	0.670		
