# Nuclear and cytoplasmic expression of survivin in 67 surgically resected pancreatic cancer patients

**DOI:** 10.1038/sj.bjc.6602826

**Published:** 2005-10-25

**Authors:** G Tonini, B Vincenzi, D Santini, S Scarpa, T Vasaturo, C Malacrino, R Coppola, P Magistrelli, D Borzomati, A Baldi, A Antinori, M Caricato, G Nuzzo, A Picciocchi

**Correction to:** *British Journal of Cancer* (2005) **92**, 2225–2232. doi:10.1038/sj.bjc.6602632

Owing to an author error, Table 2 was incorrect. The correct Table is given below.

## Figures and Tables

**Table 2:** Immunohistochemical parameters pancreatic adenocarcinoma patients

	**Number**	**%**
*Nuclear survivin expression*		
Survivin negative	42	62.7
Survivin positive	25	37.3
		
*Cytoplasmic survivin expression*		
Survivin negative	37	55.2
Survivin positive	30	44.8
		
*Cox-2 expression*		
Cox-2 negative	35	52.2
Cox-2 positive	32	47.8
		
*TUNEL staining*		
TUNEL <10	39	58.2
TUNEL >10	28	41.8

